# Extracorporeal life support for refractory cardiogenic shock. etiology and outcome in a tertiary referral hospital

**DOI:** 10.1186/2197-425X-3-S1-A755

**Published:** 2015-10-01

**Authors:** AI Hurtado-Doce, D Garcia-Saez, C Hernandez-Caballero, NJ Lees, S Ledot, PN Mohite, D Hall, AF Popov, AR Simon, C Morgan

**Affiliations:** Royal Brompton and Harefield Trust, London, United Kingdom

## Introduction

Cardiogenic shock independently of its etiology remains a clinical challenge and its mortality remains unacceptably high. Extracorporeal life support (ECLS) devices provide temporary mechanical circulatory support in patients on refractory cardiogenic shock and are usually implanted under emergency conditions in critical patients.

## Objectives

To assess etiology, indication and outcome of patients supported with veno-arterial extracorporeal membrane oxygenation (VA-ECMO).

## Methods

Retrospective observational study collecting all patients who were supported with VA-ECMO at our institution from June 2010 to March 2015. Patients who were placed on ECMO post-cardiotomy were excluded.

## Results

During the study period fifty-five patients were supported with VA-ECMO with mean age of 42 (16-66), being male 75%. The aetiology of cardiogenic shock was acute ischaemic heart disease (IHD) 10 patients (18,18%), chronic IHD with decompensated heart failure (DHF) 9 patients (16,36%), dilated cardiomyopathy with DHF 24 patients (43,63%), acute heart failure secondary to miocarditis 8 patients (14,54%) or other (pheochromocytoma, cardiac sarcoma, peripartum) 4 patients (7,27%).

Our approach to cannulation was mainly peripheral, 52 patients (94,54%). 19 patients were supported with combined IABP (35,54%), with higher incidence in the ischaemic heart disease group. Average duration of ECMO support was 7,2 days+/- 4,8 days (range: 1-20). ECMO weaning was possible in 30 patients (54,54%). Total recovery of heart function at hospital discharge was observed in 12 patients (21,81%).

16 of our patients were upgraded to long term support (Heartware HVAD^®^ System) as a bridge to transplant. During the study period 4 patients received a heart transplant.

ITU mortality was 27,27% (15 patients) and overall mortality at hospital discharge was 43,63%

(24 patients). Refractory shock with multi-organ failure was the most common cause of death

(16 patients, 29%), followed by complications related with ECMO, as uncontrolled haemorrhage

(12,72%, 7 patients).

Cardiogenic shock secondary to myocarditis represents the group with better overall survival, 100%, and IHD the one with higher mortality rate.

## Conclusions

VA-ECMO can be indicated in patients with refractory cardiogenic shock who have an underlying potentially reversible heart condition, although it can also be used as a bridge to a ventricular assist device or cardiac transplantation In-hospital survival rate of patients with VA-ECMO varies up to 50% according to the cause of the cardiac dysfunction. It remains difficult to determine which patients will benefit from VA-ECMO and when this support will be futile. Patients who require ECMO as a result of reversible causes such as myocarditis appear to have the best prognosis with higher rate of recovery. Outcome remains worst for patients with cardiogenic shock related with IHD.

